# Bulk segregant RNA-seq reveals expression and positional candidate genes and allele-specific expression for disease resistance against enteric septicemia of catfish

**DOI:** 10.1186/1471-2164-14-929

**Published:** 2013-12-30

**Authors:** Ruijia Wang, Luyang Sun, Lisui Bao, Jiaren Zhang, Yanliang Jiang, Jun Yao, Lin Song, Jianbin Feng, Shikai Liu, Zhanjiang Liu

**Affiliations:** 1 The Fish Molecular Genetics and Biotechnology Laboratory, Department of Fisheries and Allied Aquacultures and Program of Cell and Molecular Biosciences, Aquatic Genomics Unit, Auburn University, 203 Swingle Hall, Auburn, AL 36849, USA

**Keywords:** Bulk segregant analysis, RNA-seq, Disease resistance, Catfish, Allele-specific expression

## Abstract

**Background:**

The application of RNA-seq has accelerated gene expression profiling and identification of gene-associated SNPs in many species. However, the integrated studies of gene expression along with SNP mapping have been lacking. Coupling of RNA-seq with bulked segregant analysis (BSA) should allow correlation of expression patterns and associated SNPs with the phenotypes.

**Results:**

In this study, we demonstrated the use of bulked segregant RNA-seq (BSR-Seq) for the analysis of differentially expressed genes and associated SNPs with disease resistance against enteric septicemia of catfish (ESC). A total of 1,255 differentially expressed genes were found between resistant and susceptible fish. In addition, 56,419 SNPs residing on 4,304 unique genes were identified as significant SNPs between susceptible and resistant fish. Detailed analysis of these significant SNPs allowed differentiation of significant SNPs caused by genetic segregation and those caused by allele-specific expression. Mapping of the significant SNPs, along with analysis of differentially expressed genes, allowed identification of candidate genes underlining disease resistance against ESC disease.

**Conclusions:**

This study demonstrated the use of BSR-Seq for the identification of genes involved in disease resistance against ESC through expression profiling and mapping of significantly associated SNPs. BSR-Seq is applicable to analysis of genes underlining various performance and production traits without significant investment in the development of large genotyping platforms such as SNP arrays.

## Background

Performance is related to the subtle variation in gene expression and this relationship differs among individuals [1]. In well-defined families, the first level of variation comes from genetic segregation and recombination of chromosomes. As a result of segregation and chromosomal recombination, each individual has different genetic makeup. Upon a given genetic background, genetic potential carried on DNA can only be realized when the genes are expressed. At the whole genome level, expression of each gene is affected by its genetic regulatory element as well as trans-acting factors including the impact of environment. A composite of genes, transcriptional regulation, post-transcriptional modification and regulation, translational regulation and post-translational modification and regulation, along with environmental impact and genotype-environment interactions eventually determines the phenotypes of individuals. When considered at the whole genome level, expression of tens of thousands of genes and combination of these genes make the variation of performance traits extremely complex with huge variability. The task of modern agricultural genomics is to gain understanding of such variations and their relationship in determination of production and performance traits.

Traditionally, genetic and molecular biological studies are conducted to dissect these variables at different levels. For instance, the effect of alleles can be dissected through genetic and QTL mapping analysis [2-5]. Gene expression can be analyzed using high throughput methodologies such as microarrays and RNA-seq analysis [6-8]. Various epigenetic regulations have also been studied to understand the differences in gene expression with similar genetic background. Such analyses have been very powerful in determination of genetic and epigenetic factors affecting performance and production traits [9-11].

However, performance and production traits are often highly complex and the outcome of agricultural operations is affected by variations at all levels. For example, genetic background is very important because disease resistance genes allow the organism to survive the serious infections [12,13]. In most cases, disease resistance genes have been studied through genetic linkage and QTL analyses that allow the identification of genomic regions containing disease resistance genes to be identified. Even with the most powerful molecular approaches, analysis of complex traits such as disease resistance can be extremely challenging. In 1991, Michelmore et al. developed a method called bulked segregant analysis (BSA) to study disease resistance in plants [13-20]. The basic idea of BSA was that phenotypic extremes should have drastic differences in genotypes. When samples are selected from phenotypic extremes, say the best and the worst performers, and their genotypes are analyzed in bulk, a correlation of genotypes with phenotypes can be attained. In other word, the variation among individuals may be quite subtle and difficult to detect; however, the pooled samples (bulk) of the phenotypic extremes should pose a strong contrast in their genotypes at the genomic location linked to the trait. BSA has been used in numerous studies to associate phenotypes with related genomic locations.

BSA has been evolving along with various types of molecular markers including Restriction Fragment Length Polymorphisms (RFLPs) [13], Random Amplified Polymorphic DNAs (RAPDs) [14], Simple Sequence Repeats (SSRs, or microsatellites) [15,16], Amplified Fragment Length Polymorphisms (AFLPs) [16,17] and Single Nucleotide Polymorphisms (SNPs) [18]. With the development of Next-generation sequencing (NGS) technologies, BSA was first enhanced by the application of sequence-based markers such as restriction-site associated DNA (RAD) markers [21] and whole genome sequencing [22].

In recent years, the application of RNA-seq [7,8,23-27] has allowed rapid and comprehensive understanding of transcriptome level of variations. Coupling of BSA with RNA-seq has led to the development of bulked segregant RNA-seq (BSR-Seq), and it has been successfully applied in plants [28,29], but not yet demonstrated in animals. Apparently, BSR-Seq possesses the advantages of both BSA and RNA-seq, with the high throughput for deep coverage of the transcriptome as well as the strong ability to detect genetic differences underlining the traits. Such a technique is best suited to organisms with high fecundity such as many species of fish.

Catfish is the major aquaculture species in the United States, accounting for over 60% of all US aquaculture production. The two major cultivated catfish species are channel catfish *(Ictalurus punctatus)* and blue catfish *(Ictalurus furcatus)*. An inter-specific hybrid (channel catfish female × blue catfish male) has been popular for aquaculture because of strong heterosis [30]. Not only is the interspecific hybrid is popular for aquaculture, it is also a superior system to study disease resistance because of their strong phenotypic difference. Blue catfish is extremely resistant against ESC disease while channel catfish is relatively susceptible. Genetic linkage analysis of F2 generation of the interspecific hybrids [31-33] would allow identification of disease resistance/susceptibility genes. In this study, we take advantage of BSR-Seq for the analysis of disease resistant genes using the F2 generation backcross progenies (F1 hybrid backcrossed with the susceptible channel catfish) of the interspecific hybrids. Here we demonstrate that BSR-Seq is capable of 1) revealing differentially expressed genes; 2) revealing positional candidates containing genes related to disease resistance after mapping SNPs on the whole genome; and 3) revealing allele-specific expression after bacterial infection.

## Results

### Sequence assembly and analysis

RNA-seq was conducted using Illumina sequencing with three pooled samples of resistant fish, susceptible fish, and control fish. Each pooled sample contained equal amount of RNA collected from 24 individuals. Each pooled sample was barcoded such that reads from each of the three samples can be traced and analyzed separately. A total of over 400 million reads were generated with 151 million reads from the resistant fish, 116 million from the susceptible fish, and 132 million from the control fish. After quality trimming, a total of 374 million reads were carried forward for analysis, with an average read length of 95.9 bp (Table 1). Raw read data are archived at the NCBI Sequence Read Archive (SRA) under Accession SRP028159.

**Table 1:** Summary of Illumina sequencing of the catfish liver transcriptome with extreme phenotypes after ESC infection

	**Control**	**Susceptible**	**Resistant**	**Total**
Number of reads	132,406,228	116,652,262	151,256,664	400,315,154
Read length (bp)	100 bp	100 bp	100 bp	100 bp
Number of reads after trimming	123,437,613	110,344,258	140,669,530	374,451,401
Percentage kept after trimming	93.2%	94.6%	93.0%	93.5%
Average read length after trimming	95.8 bp	96.0 bp	95.8 bp	95.9 bp

The reads after quality trimming were assembled *de novo* into 232,338 non-redundant contigs (including coding and non-coding RNA) with a N50 contig length of 1,900 bp and an average contig length of 825 bp. Of the assembled contigs, over 51,000 had a length of over 1,000 bp. The assembled contigs were analyzed by BLAST searches to determine the gene identities of the contigs. Of the 232,338 contigs, 55,130 had hits to 22,126 unigenes, of which 15,599 were known genes and 6,527 were hypothetical genes (Table 2).

**Table 2:** **Summary of *de novo *** **assembly of the catfish liver transcriptome with infection of ESC generated by Illumina sequencing and assembled with Trinity**

Number of non-redundant contigs	232,338
Large contigs (≥1,000 bp)	51,601
Length of the largest contig	18,759 bp
N50 size	1900 bp
Average length of non-redundant contigs	825 bp
% reads mapped to the final reference	91.68%
Number of contigs with hits	55,130
Unigene matches	22,126
Known gene matches	15,599
Unknown hypothetical gene matches	6,527

Contigs were used as queries for BLAST searches against Zebrafish and NR protein databases. The cut-off value for gene identity was set at e-value ≤ 1e^-5^.

### Differentially expressed genes after infection

Differentially expressed genes after infection in resistant fish and susceptible fish were determined by comparing their expression levels in RPKM (reads per kilobase of exon model per million mapped reads) with that of the control group. As summarized in Table 3, a total of 224 genes were differentially expressed in the resistant group as compared with the control group. A significant fraction of the differentially expressed genes in resistant fish were highly regulated, with 42 genes (18.8%) being up- or down-regulated 10-fold or more, and 21 additional genes up- or down-regulated 5–10 fold (Table 3).

**Table 3:** **Analysis of differentially expressed genes (fold change ≥2, p ≤ 0.05) after infection with *Edwardsiella ictaluri***

	**Total**	**Up-regulated**	**Down-regulated**	**Fold change > 5**	**Fold change > 10**
Number of genes differentially expressed in resistant fish	224	130	94	63	42
Number of genes differentially expressed in susceptible fish	1,240	771	469	520	233
Regulated genes only in resistant fish	89	57	32	43	35
Regulated genes only in susceptible fish	1,093	682	411	464	200

Although a relatively small number of genes were differentially expressed in resistant fish, a large number of genes were differentially expressed in susceptible fish, with a total of 1,240 genes being differentially expressed (Table 3). Not only the number of differentially expressed genes was drastically more in susceptible fish, the number of highly regulated genes was also much greater in susceptible fish, with 233 genes being up- or down-regulated 10 folds or more, and additional 287 genes were up- or down-regulated 5–10 folds (Table 3).

### Comparison of gene expression in resistant fish and susceptible fish after infection

**Figure 1:**
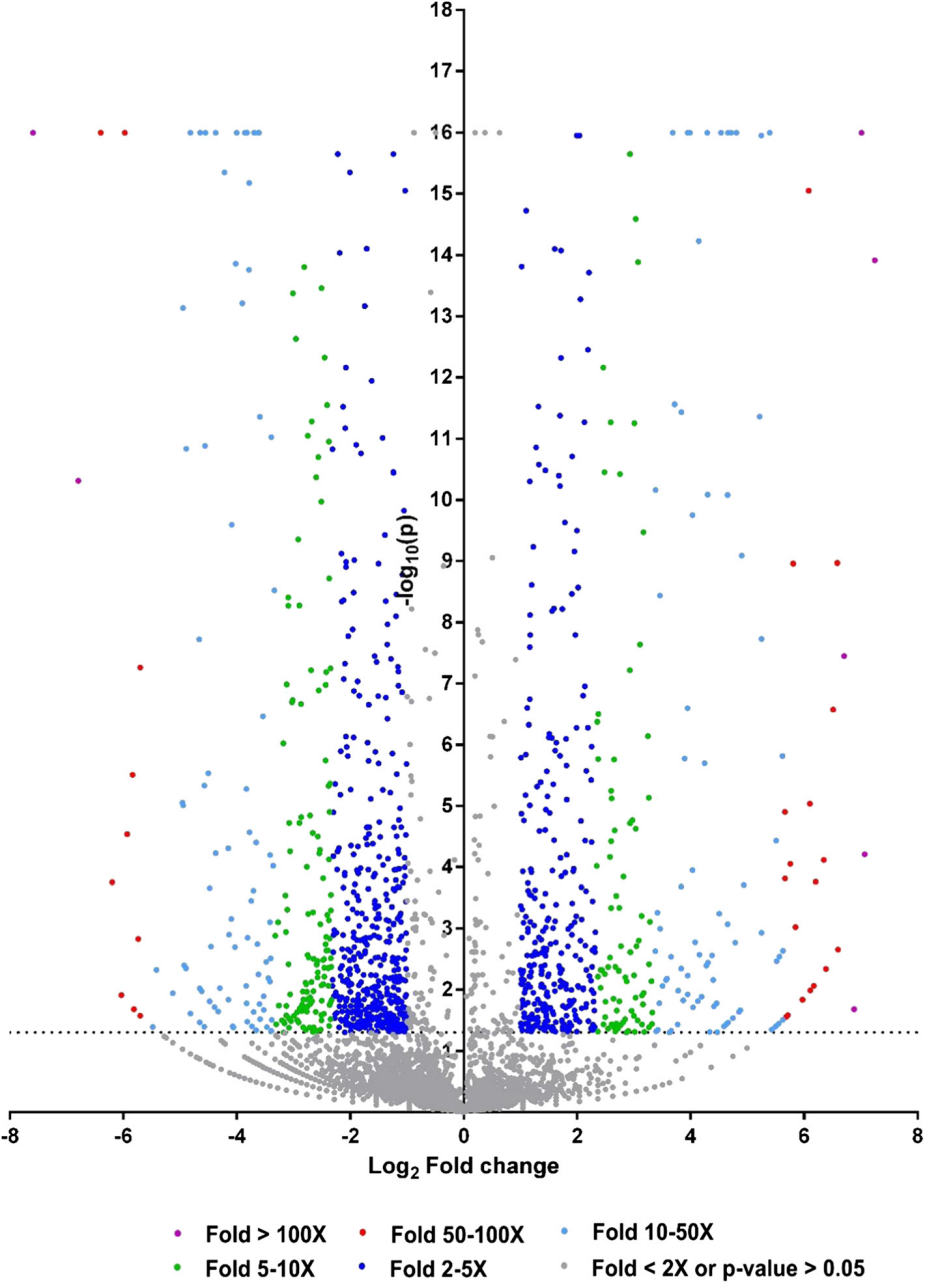
**Volcano plot of genes differentially expressed between resistant and susceptible fish.** The dots located in the positive area stand for genes expressed higher in resistant fish, and dots located in the negative area stand for genes expressed higher in susceptible fish. As shown in graphic symbol, different color were used to scale different expression fold changes; purple stands for expression fold changes higher than 100-fold; red stands for expression fold changes from 50–100 fold; light blue stands for expression fold changes from 10–50 fold; green stands for expression fold changes from 5–10 fold; blue stands for expression fold changes from 2–5 fold; and gray stands for gene expressed insignificantly (p-value > 0.05 or fold change smaller than 2).

Although a small number of genes were differentially expressed in resistant fish when being compared with the control fish, a large number of genes exhibited differential expression in resistant fish when being compared with the susceptible fish. A total of 1,255 genes were differentially expressed in resistant and susceptible fish, with 528 genes expressed significantly higher in resistant fish than in susceptible fish and 727 genes expressed significantly lower in resistant fish than in susceptible fish (Additional file 1: Table S1). Of the genes expressed significantly higher in resistant fish, 4 genes were expressed over 100 times more than in susceptible fish; 19 were expressed 50–100 times more than in susceptible fish; 86 were expressed 10–50 times more than in susceptible fish (Table 4, Figure 1). Of the genes expressed significantly lower in resistant fish, 2 genes were expressed over 100 times less in resistant fish than in susceptible fish; 10 were expressed 50–100 times less in resistant fish than in susceptible fish; 86 were expressed 10–50 times less in resistant fish than in susceptible fish (Table 4, Figure 1).

**Table 4:** **Comparison of gene expression between resistant fish and susceptible fish after infection with *Edwardsiella ictaluri***

Differentially expressed genes between resistant and susceptible fish	1,255
Gene expressed higher in resistant fish	528
>100 fold	5
50-100 fold	18
10-50 fold	86
5-10 fold	94
2-5 fold	325
Genes expressed lower in resistant fish	727
>100 fold	2
50-100 fold	10
10-50 fold	86
5-10 fold	159
2-5 fold	470

These genes are listed in Additional file 1: Table S1.

### Identification of SNPs and significant SNPs

SNPs were identified by alignment of short reads to the reference assembly of the RNA-seq. In order to be qualified for SNPs, at least 6 reads were required for each group (resistant, susceptible, or control) and a total of minor allele reads count must be greater than 3 among three groups. Using software Popoolation 2, a total of 513,371 SNPs were identified. In order to be sure the identified SNPs were properly identified, a second software package, VarScan 2 [34] was also used. A total of 482,035 SNPs were identified. The difference was caused by differences in cutoff of quality scores of sequence reads by the two programs. Although the total numbers of SNPs identified by the two software differed by approximately 6%, the vast majority (465,537 SNPs, 96.6%) of SNPs identified by the two software were identical. These SNPs were located within 31,646 contigs. In order to determine SNPs with significant difference in allele frequencies between the resistant and susceptible fish (significant SNPs), Fisher’s Exact test was initially performed. As shown in Table 5, 56,419 SNPs were identified as significant SNPs. These significant SNPs were located in 11,249 contigs. Of the 11,249 contigs, 5,480 had significant hits to known genes, and the remaining probably represented contigs assembled from 5′- and 3′-untranslated regions or from long non-coding RNAs. The 5,480 contigs with hits to known genes represented 4,304 unique genes harboring 34,584 SNPs (Additional file 2: Table S2).

**Table 5:** Identification of SNPs and significant SNPs (allele frequencies statistically different between the resistant and susceptible groups) from the assembled catfish liver transcriptome

Total number of SNPs	513,371
Number of contigs containing SNPs	31,646
Number of significant SNPs	56,419 (10.99%)
Number of contigs containing significant SNPs	11,249
Number of contigs with significant hits to genes	5,480
Number of genes containing significant SNPs	4,304
Number of genomic scaffolds containing significant SNPs	2,096

### Bulk frequency ratios

**Figure 2:**
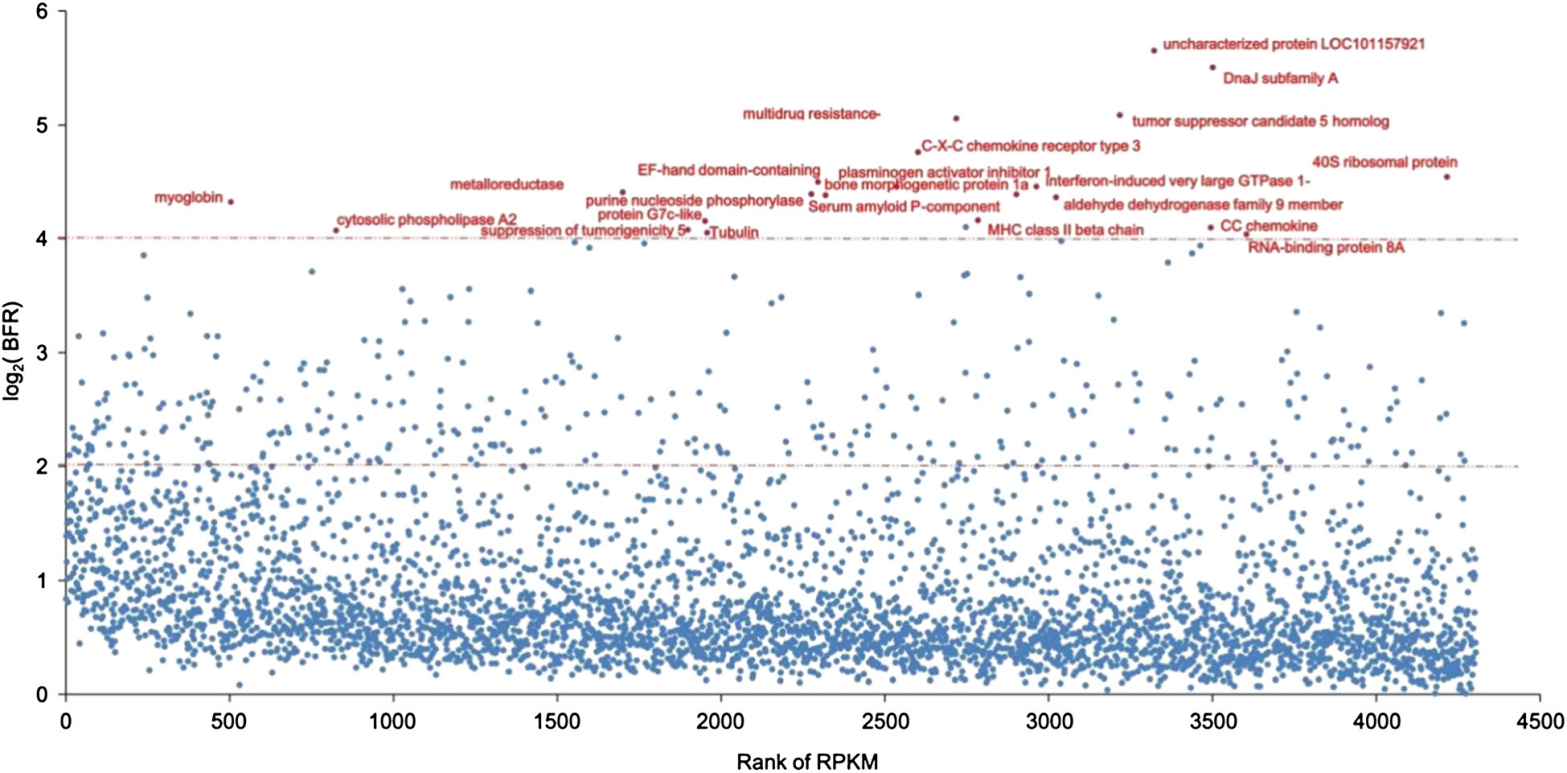
**Genes harbouring significant SNPs, plotted by the log**_**2**_**(BFR) versus rank of RPKM.** Genes which have BFR larger than 16 (log_2_ =4) were highlighted by red and their gene names were labeled.

Although significant SNPs identified through RNA-seq analysis reflect the final ratios of different alleles at the RNA level in the two bulked samples, statistical analysis for significant SNPs using Fisher’s Exact test was only the first step for screening SNPs that may be significantly associated with the trait. In order to compare the SNP allele frequencies more directly, bulk frequency ratios (BFR) were generated from the RNA-seq data between the two bulks, the resistant fish and the susceptible fish. The BFR of genes were determined by the maximum BFR of the significant SNPs generated from the Fisher’s Exact test located in this gene. As shown in Figure 2, large proportion of genes containing the significant SNPs had a BFR of at least 2. A total of 359 genes had a BFR equal or greater than 4. Among these genes, 337 (93.9%) genes had a BFR of 4–16; 23 genes had a BFR over 16; and 4 genes had a BFR over 32 (Figure 2). The four genes had the highest BFR are multidrug resistance-associated protein 5, tumor suppressor candidate 5 homolog (Interferon-induced transmembrane protein), uncharacterized protein LOC101157921, and DnaJ subfamily A member 2. The additional 19 genes with the largest BFR of over 16 are myoglobin, cytosolic phospholipase A2, metalloreductase STEAP2, suppression of tumorigenicity 5 protein, protein G7c-like, Beta-tubulin, purine nucleoside phosphorylase 4b, EF-hand domain-containing protein D2, Serum amyloid P-component precursor, plasminogen activator inhibitor 1 precursor, si:dkey-269d20.3, transmembrane protein C9orf125, MHC class II beta, bone morphogenetic protein 1a, interferon-induced very large GTPase 1-like, aldehyde dehydrogenase family 9 member A1-A, CC chemokine SCYA108, RNA-binding protein 8A and 40S ribosomal protein S3a (Figure 2). A list of the genes with various BFR values were listed in Additional file 3: Table S3.

### Genes with large BFR caused by genetic segregation

As RNA-seq data is analyzed in terms of RPKM at the RNA level, the allele ratios obtained by RNA-seq are compounded by two factors: the genotype allele frequencies at the DNA level and the relative expression levels of the two alleles at the RNA level. For instance, the two alleles may have very different genotype allele frequencies in the two bulked samples, and in these cases, even if the expression is not regulated at the transcriptional level, the final ratio of the two alleles between the bulked samples are expected to be different. However, if one of the two alleles is differentially regulated, the final allele ratio at the RNA level would be different from the allele ratio at the DNA level.

In order to differentiate SNPs with large BFR caused by genotype allele frequency difference from those with large BFR caused by allele-specific expression, the ratio of the two alleles was analyzed with combined bulk of both resistance and susceptible bulks. Theoretically, now the combined bulk should include alleles at expected segregation ratios without any connection with the phenotypes.

**Figure 3:**
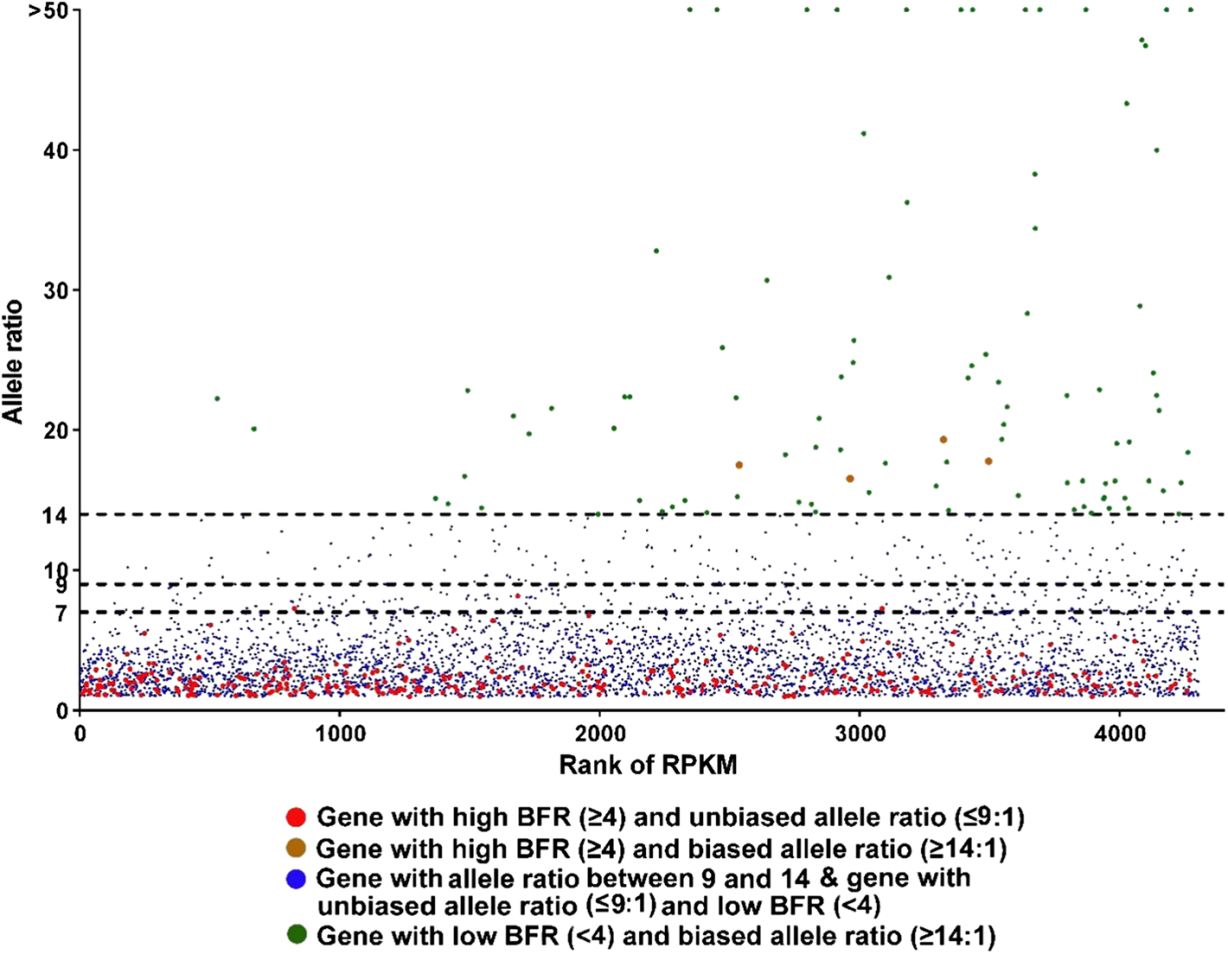
**Genes harbouring significant SNPs, plotted by their combined allele ratios versus rank of RPKM.** Red dots represent genes with BFR ≥ 4 and combined allele ratio ≤ 9; brown dots represent genes with BFR ≥ 4 and allele ratio ≥ 14; green dots represent genes with BFR < 4 and allele ratio ≥ 14, blue stands for genes with allele ratio from 9 to 14, or genes with BFR < 4 and allele ratio ≤ 9. The three threshold lines of combined allele ratio of 1:7 (maximal possible ratio at the DNA level for any polymorphic SNP with two families), 1:9 (3X maximal possible ratio at the DNA level for SNPs polymorphic in both families); and 1:14 (2X maximal possible ratio at the DNA level for any polymorphic SNP with two families) were drawn for references.

If the large BFR is caused by different allele frequencies at the DNA level in the two bulks but not by allele-specific expression, the ratio of the two alleles in the combined bulk (resistant bulk plus susceptible bulk) should be relatively small and predictable with the family structure. For instance, at an AA x AG SNP site in a single family, the progenies should have a 3A:1G allele frequency at the genomic level. However, the situation is more complex if more than one family is used. Nevertheless, the largest allele ratio at the DNA level can still be predicted. For instance, if two families are used as in this study, at an SNP site, the largest possible allele ratio at the DNA level is 7:1, i.e., AA x AG in one family, and AA x AA in the second family, where the largest possible allele ratio at the DNA level is 7A:1G. Any other combinations would result in a smaller combined allele ratio at the DNA level (Table 6). Therefore, we differentiated the large BFR caused by genetic segregation and those caused by allele-specific expression based on the combined allele ratio of the resistant and susceptible bulks. While those large BFRs with very large combined allele ratios are likely caused, at least in part, by allele-specific expression, and those large BFRs with small combined allele ratios are likely caused by genetic segregation, the BFRs in the transitional zone could be caused by both genetic segregation and allele-specific expression (Figure 3).

**Table 6:** Allele ratio combination in two families

**1st family/2nd family**	**AA xAA**	**AA x AG**	**AG x AG**
AA x AG	7:1	3:1	5:3
AG x AG	3:1	5:3	1:1
AA x AA	-	7:1	3:1

Of the 359 genes with large BFR (≥4), 347 had a combined bulk allele ratio of 7 or less. The vast majority of these had a combined bulk allele ratio of 1–3, suggesting that the large BFR of these genes were not caused by allele-specific expression, and likely caused by genetic segregation.

### Genes with large BFR caused by allele-specific expression

As shown in Figure 3, a large number of genes harboring significant SNPs had a significantly higher combined allele ratio, with 286 genes had an allele ratio of greater than 9:1 (genes above the threshold of 9 in Figure 3). Considering that most of the genes with BFR >4 had a combined allele ratio of 1–3 (see above and Figure 3), many of the genes with combined allele ratio of greater than 9 could be caused by allele-specific expression. On the cautious side, even in the extreme cases of 7:1 allele ratios at the DNA levels, twice the largest possible allele ratio at the DNA level should be 14:1. As shown in Figure 3, after the fisher’s exact test on the significant different level between the two alleles on the genes with allele ratio ≥ 14, 98 genes had a combined allele ratio of greater than 14 with FDR p-value smaller than 0.05, indicating that these large allele ratios are caused, at least in part, by allele-specific expression. A list of these allele-specific expressed genes was provided in Additional file 4: Table S4. Of the 98 genes with high combined allele ratios, 4 genes were with BFR higher than 4 and allele ratio higher than 14. They are plasminogen activator inhibitor 1, interferon-induced very large GTPase 1-like, uncharacterized protein LOC101157921 and CC chemokine SCYA108. Apparently, these large combined allele ratios were caused both by genetic segregation and by allele-specific expression.

### Location of genes with high bulk frequency ratio (BFR)

To determine the genomic location of SNPs with high levels of BFR, genes containing SNPs with high BFR (BRF ≥ 4) were initially used as query for BLAST searches against the draft catfish genome sequence scaffolds in relation to the linkage map. A total of 354 genes were identified to contain significant SNPs with high BFR (Additional file 3: Table S3). BLASTN searches were conducted to determine the locations of the 354 genes on the scaffolds of the catfish genome draft sequence (unpublished data), and they were found to be within 201 genomic sequence scaffolds. Of the 201 scaffolds, 134 can be located in linkage groups, and the remaining cannot be located on linkage groups because no markers from these scaffolds were mapped.

**Figure 4:**
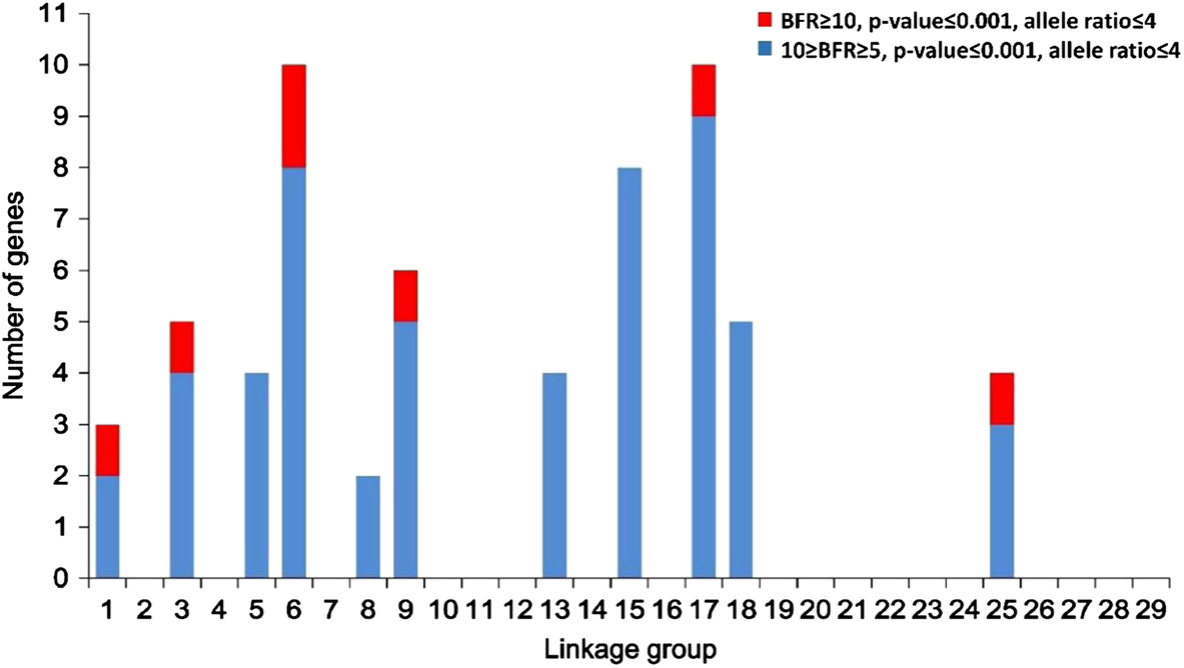
**The distribution of high BFR genes (BFR ≥ 4) on linkage groups.** Blue stands for the number of genes with 5 ≤ BFR < 10, red stands for the number of genes with BFR ≥ 10.

In order to further analyze the linkage disequilibrium (LD) and to reduce the possibility of false positives, we placed additional stringent parameters including SNPs with BFR ≥5, along with combined bulk allele ratio <4 (to assure that the high BFR is likely caused by genetic segregation) and the FDR adjusted p ≤ 0.001 for significant SNPs. As shown in Figure 4, eleven LG potentially harbor genes with significant SNPs. However, with only two families used in the study, if a gene is truly involved in resistance, long stretches of genomic segments are expected to be in LD because of genetic linkage. Therefore, the locations and distributions of genes containing significant SNPs with the following characteristics were further analyzed: 1) at least 5 genes were involved in the LG with significant SNPs; 2) or if the genes with significant SNPs were fewer than 5, at least one SNP has a BFR equal or greater than 10. Using this set of criteria, eight LG appeared to harbor QTLs involved in ESC disease resistance. These LG were LG 1, 3, 6, 9, 15, 17, 18, and 25. Of these eight LG, six contained genes with SNPs having BFR > 10 (Figure 4). Of the eight LGs, LG6, 15, and 17 had the largest numbers of genes with significant SNPs.

**Figure 5:**
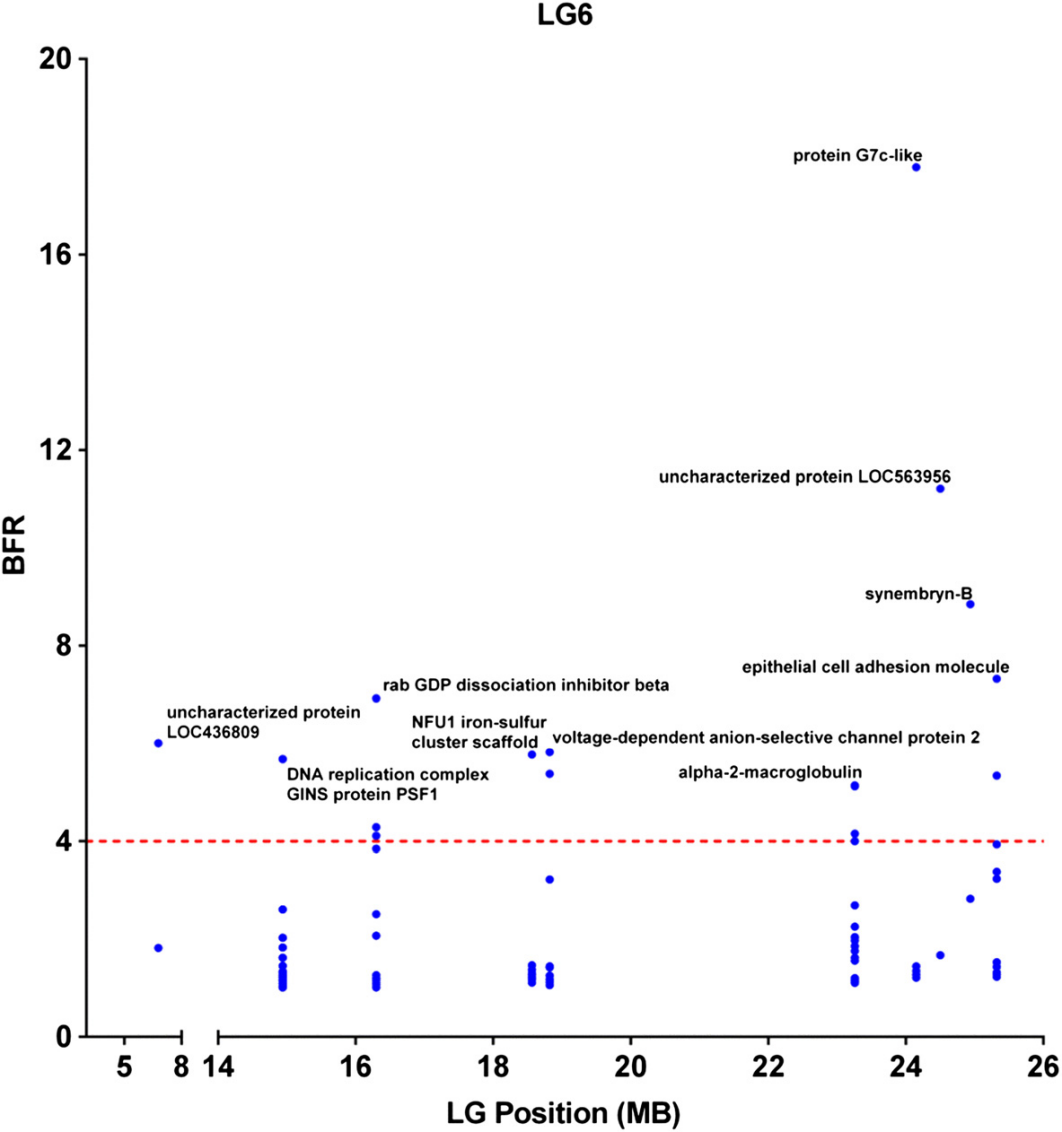
Distribution of genes containing SNPs of high BFR in linkage group 6.

**Figure 6:**
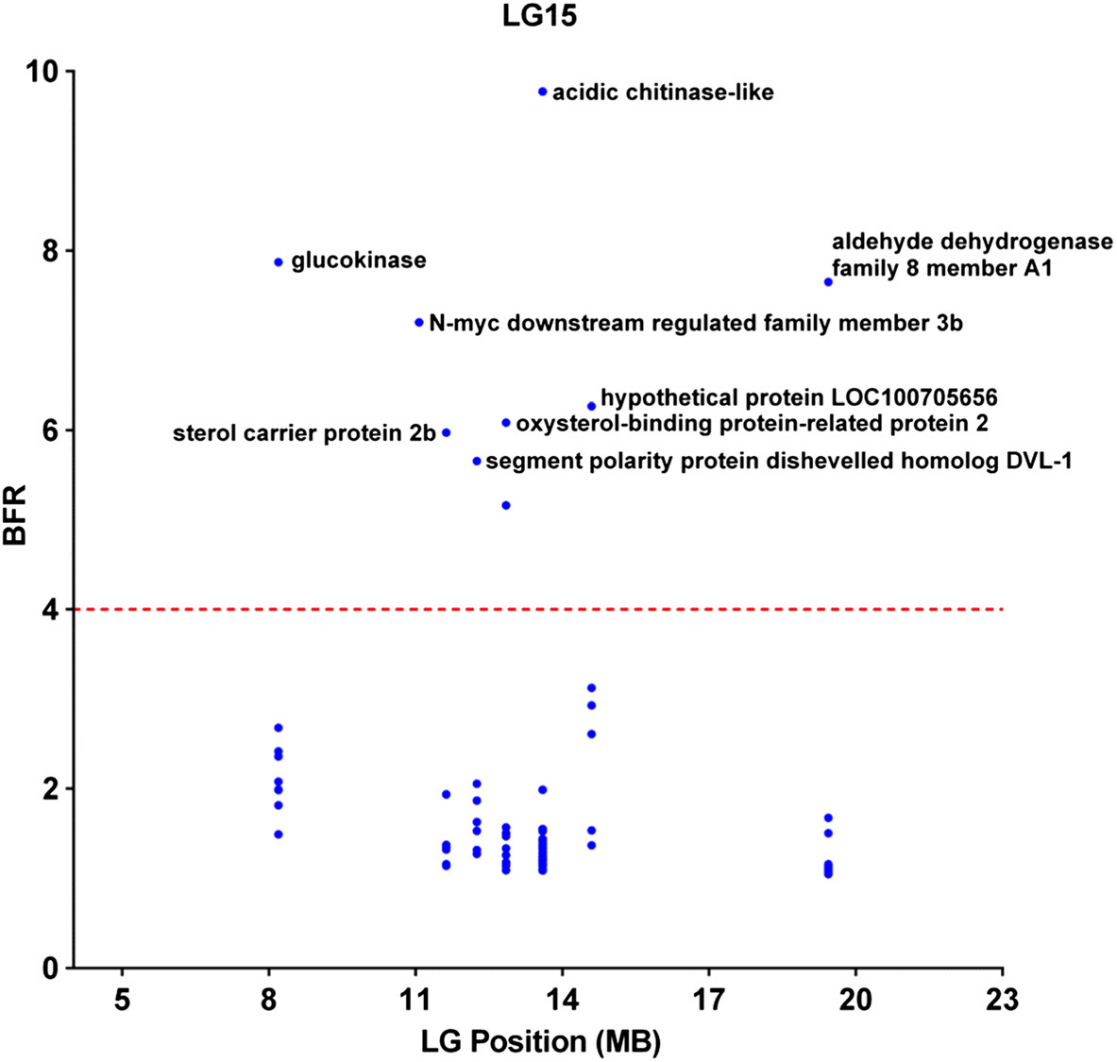
Distribution of genes containing SNPs of high BFR in linkage group 15.

**Figure 7:**
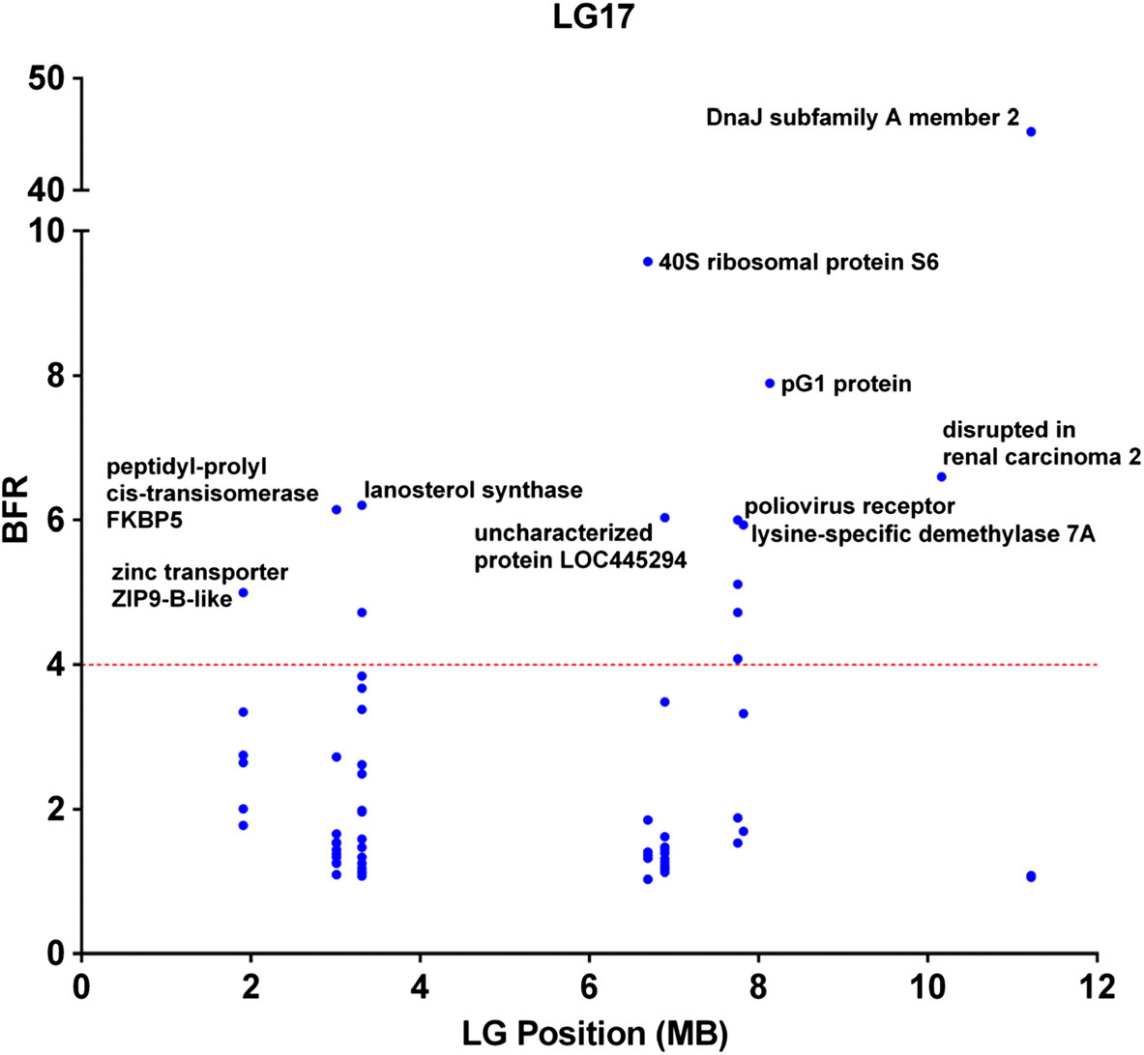
Distribution of genes containing SNPs of high BFR in linkage group 17.

Detailed distributions of genes within LG were determined by locating the genes on the scaffolds along the linkage group. As shown in Figure 5, in LG6, SNPs within the von Willebrand factor A domain-containing protein 7-like (G7c-like) gene had a very high BFR (17.8), and the nearby genes had a much lower BFR, suggesting that the resistant gene(s) were near the G7c-like gene. In LG 15, the SNP with the highest BFR was found in the middle of LG15 within the acidic chitinase-like gene, and BFR values in surrounding genes were gradually lower along both sides of the LG, suggesting tight linkage of the resistance gene(s) near the acidic chitinase gene (Figure 6). In LG17, one SNP was found to have extremely high BFR (45.3) within the DnaJ subfamily A member 2 gene (homologue of HSP40). BFR values of SNPs within genes left of the DnaJ subfamily A member 2 gene dropped sharply, but no genes with high BFR values were found on the right side of the DnaJ subfamily A member 2 gene (Figure 7).

### Parental origin of highly expressed alleles

**Figure 8:**
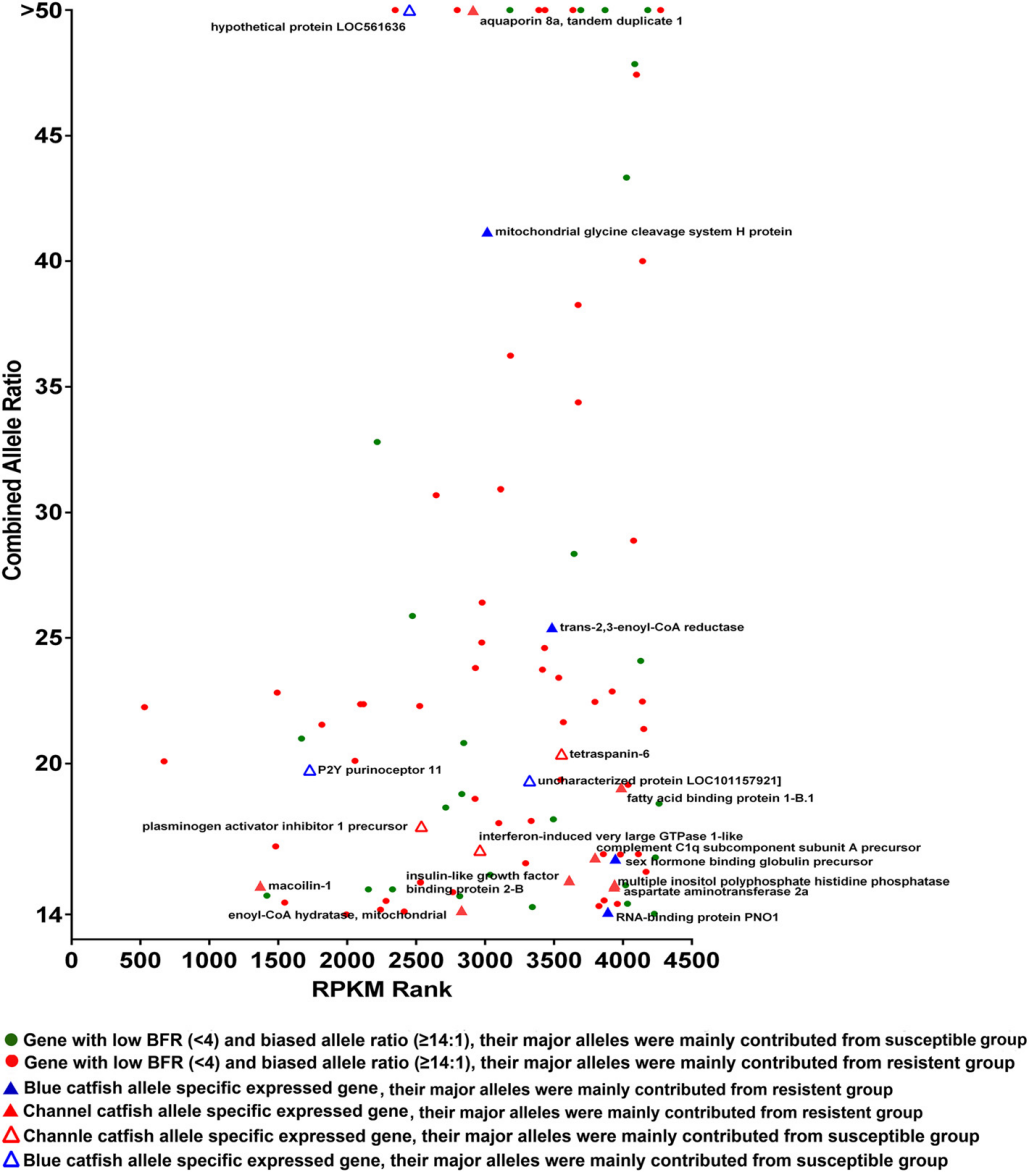
**Genes harbouring significant SNPs, plotted by their combined allele ratios versus the rank of RPKM.** Red dots stand for genes with the preferentially expressed allele expressed higher in resistant group and their parental origin unknown; Solid red triangles stand for genes with the preferentially expressed allele expressed higher in resistant group and their parental origin being channel catfish; Unfilled red triangles stand for genes with the preferentially expressed allele expressed higher in susceptible group and their parental origin being channel catfish; Solid blue triangles stand for genes with the preferentially expressed allele expressed higher in resistant group and their parental origin being blue catfish; Unfilled blue triangles stand for genes with the preferentially expressed allele expressed higher in susceptible group and their parental origin being blue catfish; and green dots stand for genes with the preferentially expressed allele expressed higher in susceptible group and their parental origin unknown.

As blue catfish is generally more resistant to ESC disease than channel catfish, an interesting question to ask is which allele was preferentially expressed after ESC infection within the backcross progenies used in this study. We therefore attempted to analyze the parental origin of the alleles for the genes with high combined allele ratios. As shown in Figure 8, a total of 98 genes harboring SNPs with combined allele ratio of 14 or greater were identified. Of these, the parental origins could be determined for 18 genes with existing genome information, while the parental origin of the remaining 80 could not be determined. Of the 18 genes, 11 were of channel catfish origin and 7 were of blue catfish origin. Of the 11 genes preferentially expressed with channel catfish alleles, six were expressed high in resistant fish and five were expressed highly in susceptible fish. Similarly, of the 7 genes preferentially expressed with the blue catfish alleles, four were expressed highly in resistant fish while 3 were expressed highly in susceptible fish (Figure 8).

## Discussion

In this study, we conducted BSR-Seq [28,29] by combining the NGS-based RNA-seq [25] with bulk segregant analysis [35] for the analysis of genes involved in disease response and disease resistance against enteric septicemia of catfish (ESC). Such a simple combination of RNA-seq and BSA analysis allowed identification of differentially expressed genes, localization of disease resistance-related genes in linkage groups by mapping SNPs on the whole genome, and analysis of allele-specific expression.

BSR-Seq carried the full capability of RNA-seq that allowed identification of differentially expressed genes. Comparison of expression in resistant fish pool and susceptible fish pool with the control allowed the identification of differentially expressed genes after infection. A total of 1,240 and 224 genes were identified to be differentially expressed after ESC infection in susceptible fish and resistant fish, respectively. In the susceptible fish, many of the up-regulated genes represent the acute phase response protein genes, as previously reported [36,37]. Apparently, microarray studies were limited to the gene probes existing on the array, while RNA-seq analysis has the ability to detect all induced genes, depending on their expression levels. Clearly, the greater numbers of genes identified from this study after infection indicated that RNA-seq is more sensitive than the microarray analysis. Although RNA-seq analysis was also previously conducted after ESC infection [38], the tissues were different in these studies. In the work of Li et al. [38], intestine tissue was used, while liver was used in this study. Nevertheless, many of the differentially expressed genes identified here in the liver were among the differentially expressed genes in the intestine, as well as those identified in the microarray studies. For instance, the acute phase response (APR) genes such as CC chemokines, Toll-like receptors, complement component proteins, catechol-O-methyltransferase domain containing 1, apolipoprotein proteins, fibrinogens, angiotensinogen, MHC class I and II, Tumor necrosis factors were all found to be up-regulated, as found previously [36,37].

The use of phenotypic extremes, resistant and susceptible fish, allowed comparison of expression patterns of genes involved in immune responses, although the time point was quite different. For instance, a number of immune-related genes were found to be expressed higher in resistant fish than in susceptible fish including apolipoprotein A IV, apolipoprotein Ab, apolipoprotein Eb, apolipoprotein Bb, apolipoprotein B100, apolipoprotein M and complement component 1q (C1q), complement component 1 s (C1s), complement component 3, complement component 3a, fibrinogen alpha, fibrinogen gamma, MHC class I, and MHC II. These genes expressed at higher levels in resistant fish could indicate their importance in the related disease resistance. Apolipoproteins have been shown to be important for disease resistance in mice [39-41] and chickens [42,43].

Apparently, a much larger number of differentially expressed genes were identified in susceptible fish than in resistant fish as compared with the control. We believe that much of this difference was caused by the different sampling time (3–5 days versus 2 weeks) between these two groups. As heavy mortalities occurred 4–6 days after infection, the vast majority of differentially expressed genes in susceptible fish represented the massive responses of the host against the infection including the acute phase proteins and genes involved in inflammation and immune responses [44,45]. In contrast, the resistant fish samples were collected two weeks after infection from the survivors. As such, these resistant fish were either “resistant” or not infected, or may have cleared the bacteria from their system. The massive host responses to infection may have been over by the time of two weeks after infection.

**Figure 9:**
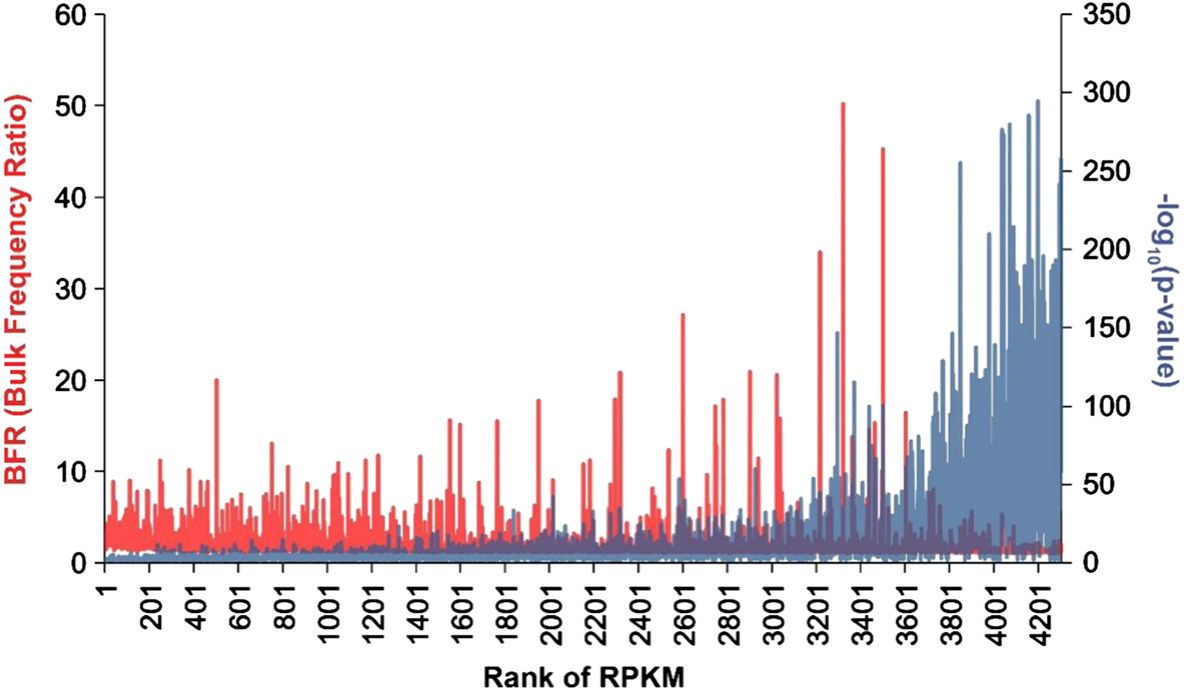
**Relationship between RPKM, BFR and p-value.** The BFR is shown on the left-Y axis (red) and P-values are shown on right-Y axis (blue), both against the rank of RPKM on X-axis. Note there is correlation between p-values and RPKM, but not the BFR.

In addition to its ability to identify differentially expressed genes, BSR-Seq carried the ability to identify significant SNPs between the pools of very strong phenotype contrast. Localization of the significant SNPs along the chromosomes should allow identification of positional candidate genes responsible for the trait. In this study, use of F2-generation backcross progenies, when coupled to pooling of samples from phenotypic extremes, allowed analysis of significant SNPs between the resistant and susceptible fish. We initially identified a set of significant SNPs between the two bulks using Fisher’s Exact test. It’s the first step to remove the SNPs not relevant to the target trait in this study, by setting the FDR adjusted p-value ≤0.05 as the cutoff to identify the significant SNPs and reduce the computation intensive in the downstream analysis. And the BFR of the SNPs with FDR adjusted p-value > 0.05 were from 1 to 2.38, which indicated that non-significant SNPs won’t affect the assignment of gene-level BFR. Although Fisher’s Exact test is technically suitable for the binary SNP markers, it is compromised by the expression levels. Because the “allele frequencies” were called at the RNA level, highly expressed genes had much lower p-values (Figure 9). Therefore, p-values were used only as the initial step for the identification of positional candidate SNPs linked with resistance QTLs.

One gene can harbor many SNPs, but not all of them are relevant to disease resistant. The low BFR of a SNP means the allele frequency of that SNP is similar between the susceptible group and resistant group, and such SNPs are irrelevant SNPs in relation to disease resistance. As to the fact that different SNPs within a single gene can have different BFR, there may be several explanations including: 1) although the number of sequences from each allele at an SNP site should be proportional to the “allele frequency”, that may not be the case practically, simply because the sequencing depth is limited; 2) We are dealing with pooled samples from two families that were derived from interspecific hybrids. Therefore, there are SNP sites that are polymorphic only within channel catfish, only within blue catfish, polymorphic in both channel catfish and blue catfish, or not polymorphic within channel catfish or blue catfish, but are polymorphic between the two species. Thus allelic ratios at different SNP sites are expected to be different. Given such complexities, it is reasonable to use SNPs with the highest BFR within each gene. In contrast, either use of median or mean BFR or use of all the SNPs for each gene may bring irrelevant SNPs into the consideration for the analysis of SNP localization, which can lead to the underestimate of the BFR in the candidate region or even miss the candidate region due to the incorrect decay pattern of LD. In addition, it is worth to mention that we used pooled sample for the BSR-seq, which could induce the difficulty to the assessment of the within-group variance, however, this is the innate limitation of the Bulked segregant analysis. Currently, there is no optimal resolution to avoid this limitation, but some studies claimed that this flaw will not cause a serious bias in the pooled sample RNA-Seq analysis [7].

Bulk frequency ratio (BFR) was previously used as an effective parameter for genetic analysis in BSA or BSR-Seq [28]. However, in those cases, genotypes were determined using DNA. Here in this study, the “allele frequency” was calculated from the mapped reads of RNA samples, and thus the calculated BFR could be compromised by allele-specific expression. In order to identify positional candidates for resistance using transcriptome datasets generated from BSR-Seq, we need to differentiate allele frequencies caused by genetic segregation and those caused by allele-specific induction/suppression: Significant SNPs with large BFR and small combined allele ratio are likely to be caused by genetic segregation; significant SNPs with small BFR but large combined allele ratio are likely caused by allele-specific expression, while significant SNPs with large BFR and large combined allele ratios may have been caused by both genetic segregation and allele-specific expression. For instance, if the allele ratio at an A/G SNP site is 10 to 1 in resistance pool, and 1 to 10 in susceptible pool, the BFR should be 10. When the two bulks were combined, now the allele ratio of A/G is 11:11 = 1. This SNP, with a high BFR and a low combined allele ratio, should be a SNP with allele frequency difference between the bulks caused by genetic segregation. In contrast, when one of the two alleles is differentially up-regulated, the combined allele ratio will stay large. For instance, at an A/G SNP site, if A is significantly up-regulated in the resistant fish, say 100A:5G, and in susceptible fish, A and G are roughly expressed equally, both at low levels, say 5A:5G. In this case, the BFR = (100/105)/(5/10) = 1.91; when the two bulks are combined, the combined allele ratio would be 10.5. Apparently in this case, the large allele ratio is caused by allele-specific expression.

In this study, each bulk was made of 24 fish with 12 fish from one family and 12 fish from a second family. As the exact allele ratio at each SNP site is unknown in the two families, we made several assumptions for the analysis of SNPs due to genetic segregation and those due to allele-specific expression. At an A/G SNP site, the parent in one family could be AA x GG, AA x AG, or AG x AG. In these cases, the largest allele ratio can be 3:1 (in the case of AA x AG) at the DNA level. When two families were used, as in this study, the largest allele ratio at the DNA level could be 7:1, i.e., AA x AG in one family, and homozygous AA x AA in another family. Any SNPs with significantly larger combined allele ratio than 7:1 would suggest allele-specific expression. We identified SNPs with combined allele ratio of greater than 14 (twice the largest possible allele ratio at the DNA level) as being allele-specifically expressed. Apparently, many SNPs fell between the two possibilities, and to obtain reasonable assessment of those caused by genetic segregation and those caused by allele-specific expression, we neglected those ones between the two possibilities (Figure 3).

True linked SNPs are characteristic in that many significant SNPs can be observed in nearby genomic locations because of genetic linkage. In catfish, the whole genome sequence assembly is still under way. We therefore, mapped the significant SNPs to scaffolds and then examined the patterns of the SNP distribution. Within a QTL region, statistical significance should be the highest with the gene underlining the performance trait, and LD should decay gradually on both sides of the chromosome surrounding the gene [44]. In our study, quite many significant SNPs were located on unmapped scaffolds, but many were also mapped to genetic linkage groups including LG6, LG15, and LG17 that included at least 10 genes with high BFR (≥5) and low combined allele ratio (≤4). As shown in Figures 5, 6, and 7, the LD appeared to be decaying around the most significant SNPs, suggesting that these genomic regions indeed contain resistance-related genes. For instance, in LG6, the gene containing the most significant SNP was protein G7c-like gene located at the 24 Mb position, and the BFR values on both sides of this gene were significant, but lower than BFR within the G7c-like gene (Figure 5). Similarly, the gene containing the highest BFR was acidic chitinase-like gene that located in the middle of a 23 Mb DNA in LG15, and the BFRs were lower on both sides along this LG (Figure 6). In LG17, the SNPs with highest BFR was located within the DnaJ subfamily A member 2 gene close to the end of the 12 Mb DNA in LG17, and the BFR on both sides were lower (Figure 7). It was unknown if the detected genes with the highest BFR were themselves involved in disease resistance. This was because some linked genes with even greater BFR were not yet mapped to the linkage group, staying as isolated scaffolds, and therefore cannot be viewed under the same “Manhattan plot”, or because the expression level of the real disease resistance gene was so low that it was not detected under the BSR-Seq analysis.

One logical thought is that if one gene is truly involved in disease resistance, it should be correlated with positional candidate genes as well as expression candidate genes. In other words, it should be differentially expressed either between resistant and susceptible fish or differentially expressed after infection, and located at a genomic location the resistance phenotype is mapped. A cross examination of differentially expressed genes and genes with high BFR but low combined allele ratio allowed identification of 17 genes (Table 7). Only four of these 17 genes have been mapped to linkage groups, whereas the remaining 13 were just mapped to isolated scaffolds. Further mapping studies are required to determine how many QTLs are involved. These genes included some important immune function related genes such as the NLR, MHC-related genes, and Mannan-binding lectin serine peptidase. Regardless of their direct involvement in the disease resistance, the importance of these genes in resistance and immune responses to ESC should not be neglected. Additional research is warranted to determine if these genes are responsible for the resistance against ESC.

**Table 7:** Differentially expressed genes after infection with high bulk frequency ratios and low combined allele ratio

**Gene**	**BFR**	**Combined allele ratio**	**Known function**
Metalloreductase STEAP2	21.2	3	Regulates iron ion homeostasis and involved in endocytosis
Tumor suppressor candidate 5 homolog	34.0	2.3	Inhibits breast tumor formation in *vivo*
Protein G7c-like	17.8	3.2	Effect the susceptibility to lung tumors
Acidic chitinase-like	9.8	1.4	Participates in the defense against nematodes, fungi and bacteria. Plays a role in T-helper cell type 2 (Th2) immune response.
Mannan-binding lectin serine peptidase 2	4.1	2.2	Lectin complement pathway actication
Inter-alpha-trypsin inhibitor heavy chain H4	4.3	1.3	Type II acute-phase protein (APP) involves in inflammatory responses to trauma. May also play a role in liver development or regeneration.
D-amino acid oxidase	4.3	1.8	Regulation of the glutamatergic neurotransmission; may play a role in the glutamatergic mechanisms of schizophrenia
MAWD binding protein like	4.4	1.4	Inhibits proliferation and invasion in gastric cancer
Cytokeratin-like	4.5	1.5	Mediate epithelial innate defense through their antimicrobial properties
Spermidine/spermine N1-acetyltransferase	4.7	1	Involves in polyamine homeostasis
Stonustoxin subunit alpha-like	4.7	1.9	Induces hemolytic activities, displays edema-inducing activities, increases vascular permeability and interferes irreversibly with neuromuscular function.
UDP-glucose 4-epimerase	4.8	1.8	Catalyzes the epimerization of UDP-glucose to UDP-galactose and the epimerization of UDP-N-acetylglucosamine to UDP-N-acetylgalactosamine.
Major histocompatibility complex class I UDA	5.1	3.1	Play an important role in immune response and antigen processing and presentation
NLR-C8	5.9	1.4	Involves in the gram negative bacteria recognition. Against the intracellular pathogen.
MARCKS-like 1a	6.1	2.5	Most prominent cellular substrate for protein kinase C. It can bind calmodulin, actin, and synapsin.
3-oxoacid CoA transferase 1a	7.0	1.3	Key enzyme for ketone body catabolism. Also plays and important roles in the energy metabolism of spermatozoa.
MHC class II beta chain	17.9	2.4	Involves in antigen processing and presentation of peptide or polysaccharide antigen via MHC class II

A total of 98 genes were identified as genes with allele-specific expression (Figure 3). One obvious question is what causes allele-specific expression (ASE). Two hypotheses were previously proposed to account for ASE [45,46]. In the first hypothesis, mutations in cis-acting DNA elements can cause differences in binding of trans-acting factors, especially when such mutations are located in the promoter or enhancer regions. Inversely, mutations in the trans-acting factors would also cause their differences in binding to their target sequence. In the second hypothesis, epigenetic factors such as differential methylation of the two alleles can cause differences in expression levels. It has long been known that mutations in non-coding regions which affect gene expression can cause human genetic disease [45,47]. A differentially expressed gene exhibits *cis*-acting variation when the differential expression is caused by factors linked to the differentially expressed alleles, such as differences in promoter sequences or chromatin state. The list of examples in which *cis*-acting regulatory variation plays a key role in phenotypic variation are increasing [45]. *In vitro* experiments prove that variants in gene promoter regions effect rates of transcription and these variants may also lead to a significant proportion of differential allelic expression [46]. In addition, expressed genes contain *trans*-acting variation when the differential expression is caused by factors unlinked to the differentially expressed alleles, such as differences caused by genetic background and regulatory networks.

The importance of DNA methylation as a driving factor in allele specific expression has been claimed and proved by a number of studies [46,48]. In these studies, a direct correlation between allele specific expression and methylation was observed. Clearly, the different epigenetic state of each haploid genome is a major factor in the expression of the two alleles. Although X-chromosome inactivation and silencing are usually considered to be mainly related to epigenetic effect [49], some studies also suggested that the change of gene regulation caused by epigenetic modification of sequence variation might be a common pathogenic mechanism in mammals [50]. One hypothetical role for epigenetics is genetic imprinting leading to mono-allelic expression [51]. Both *in vivo* and *in vitro* experiments indicate that allele-specific differences in the rate of transcription are common existed, if not all genes are likely to show differential allelic expression in different individuals [46]. However, the role of ASE genes in complex traits is still not clear.

Most ASE studies have been conducted in humans, and no studies have been conducted in fish. In addition to the above discussed possibilities, it is noteworthy that we used an interspecific hybrid system in this study. In the channel catfish backcrossed progenies, overall 50% of chromosomes are “homozygous” from channel catfish while 50% chromosomes are heterozygous with one chromosome being from channel catfish and the other from blue catfish. If a *trans*-acting factor is transcribed from channel catfish genes, it would bind the *cis*-acting elements from channel catfish with greater affinity, causing allelic expression. This could be another explanation for the observed ASE.

The significance of the observed allele-specific expression in relation to phenotype is unknown at present. To date, the majority of expression analysis focus on the total amount of the transcripts. However, emerging evidence underlies the importance of understanding the allele specific transcript in cases of disease [45,52]. In our studies here, among the 98 allele-specifically expressed genes, parental origin of alleles can be determine for only 18 genes. Among these 18 genes, the channel catfish allele was expressed higher in 11 genes (six high in resistant fish and five high in susceptible fish), and blue catfish allele was expressed higher in 7 genes (four high in resistant fish and three high in susceptible fish). There was no correlation of resistance with a specific parent. However, it is possible that certain combinations of alleles would warrant resistance and certain combinations of alleles would lead to susceptibility. This clearly warrants future studies.

## Conclusions

In this study, we demonstrated the application of BSR-Seq to study disease resistance by combining RNA-seq with bulk segregant analysis. It has the full capacity for the identification of differentially expressed genes, the capacity to identify significant SNPs between phenotypic bulks, the capacity to potentially identify the positional candidate genes, and the ability to identify allelic expressed genes. Among many differentially expressed genes, 17 genes were also among the genes that had high BFR and low combined allele ratio, suggesting that these genes could be potentially involved in disease resistance.

## Methods

### ESC bacterial challenge

All procedures involving the handling and treatment of fish used during this study were approved by the Auburn University Institutional Animal Care and Use Committee (AU-IACUC) prior to initiation. Four families of backcross progenies (average size 35 ± 1.3 g) were reared at the Auburn University Fish Genetics Research Unit prior to challenge. Fish were challenged in 500 L (400 L water) aquaria with control group containing 400 fish (100 per family) and treatment group containing 1200 fish (300 per family). The MS-S97-773 isolate of *E. ictaluri* bacteria was obtained from a natural outbreak and utilized in the experimental challenge. Bacteria were re-isolated from a single symptomatic fish and biochemically confirmed by appearance (small, punctate white colonies) and through biochemical assay (oxidase negative, fermentative in O/F glucose or glucose motility deeps (GMD), triple sugar iron (TSI) slant reaction K/A with no H_2_S, and negative for indole production in tryptone broth). The confirmed bacteria were then cultured in Brain Heart Infusion broth (BHI) and incubated in a shaker incubator at 28°C overnight. The concentration of the bacteria was determined using colony forming unit (CFU) per mL by plating 10 ml of 10-fold serial dilutions onto BHI agar plates. During challenge, 1000 mL bacterial culture with a concentration of 4 × 10^8^ CFU/ml was added into the aquaria. Water was turned off in the aquaria for 2 h of immersion exposure, and then continuous water flow-through resumed for the duration of the challenge experiment. Control group was treated with same volume of brain heart infusion (BHI) medium at the same time. During 3–5 days after challenge, all dying fish with classical ESC clinic signs were collected as susceptible fish from two families. After two weeks of the challenge, all survival fish were collected as resistant fish from the same two families. Also, the fish in control group were collected at that time. The fish were euthanized with tricaine methanesulfonate (MS 222) at 300 mg/l before tissue collection.

### Sampling and RNA isolation

Equal amount of liver tissue was used from each of the 72 fish (12 fish/family, 24 fish/group each for resistant, susceptible, and control) used for RNA isolation. The tissue samples were ground separately to a fine powder in the presence of liquid nitrogen. Total RNA was extracted using the RNeasy Universal Tissue Kit (Qiagen, USA). The samples belonging to the same group were then diluted to the same concentration and pooled together prior to library construction.

### Illumina sequencing

Sequencing libraries were prepared with 2.14-3.25 μg of starting total RNA and processed using the Illumina TruSeq RNA Sample Preparation Kit, as dictated by the TruSeq protocol. The library were amplified with 15 cycles of PCR and contained TruSeq indexes within the Illumina adaptors, specifically indexes barcode 1–3 to label the three groups. The final, amplified library yields were 30 μl of double-stranded product (19.8-21.4 ng/μl) with an average length of 275 base pair (bp), indicating a concentration of 110–140 nM. After quantitation performed using KAPA Library Quant Kits (Kapa Biosystems, USA) and dilution, the library were clustered 3 in one lane and sequenced on a Hiseq 2500 instrument with 100 bp paired end (PE) reads at the HudsonAlpha Genomic Services Lab (Huntsville, AL, USA). The image analysis, base calling and quality score calibration were processed using Illumina Pipeline Software v1.5, and FASTQ reads files containing the sequencing read, quality scores and paired reads information were exported for the following trimming and assembly process. Raw reads were processed for initial trimming by CLC Genomics Workbench (version 5.5.2; CLC bio, Aarhus, Denmark). Adaptor sequences, ambiguous nucleotides (’N’ in the end of reads), and low quality sequences (quality scores < 30 or read length < 30 bp) were removed.

### *De novo* assembly

As the primary algorithm used in RNA-seq assembly, the *de bruijn graph* method was utilized in this study. Trinity version 2013-02-25 was chosen in this study due to its good performance [53]. Briefly, the raw reads were assembled into unique sequences of transcripts in Inchworm via greedy K-mer extension (k-mer 25). After mapping of reads to Inchworm contigs, Chrysalis incorporated reads into *de bruijn* graphs and the Butterfly module processed the individual graphs to generate full-length transcripts. And then CD-hit [54] and CAP3 [55] were used to remove assembly redundancy by setting global sequence identity in CD-hit to 1, the minimal overlap length and percent identity in CAP3 to 100 bp and 99%.

### Gene identification and annotation

The final Trinity assembly contigs were used as queries against the NCBI non-redundant (nr) protein database and the zebrafish protein database using BlastX by setting the cut-off value (E-value, the likelihood that the matching sequence is obtained by chance) of 1e^-5^ and returning only the top 10 hit results of each query. The top gene identifications and names were initially assigned to each contig. “Hypothetical” or “uncharacterized” top BLAST results were replaced by more informative hits from the top ten lists when available.

### Transcript-level gene expression analysis of different groups

The modified assembly of Trinity including all unigenes with blast hits was used as the pseudo-reference against which trimmed reads were mapped for gene expression analysis. Reads per kilobase of exon model per million mapped reads (RPKM) [56] were calculated as the original expression value. The original expression values were scaling normalized in order to ensure that samples were comparable [57]. The expression fold change was calculated based on the modified expression value between the infection and control groups. The Kal’s test [58] was used to test the significance of the expression fold change. The initial p-value were adjusted by False discovery rate (FDR) method [59]. Analysis was performed using the RNA-seq module and the expression analysis module in CLC. The threshold of gene expression selection was set to: FDR adjusted p-value <0.05, mapped reads >5, weighted proportions of fold change ≥ |2|, or unless otherwise clarified for more stringent analysis.

### Sequencing mapping and significant SNP identification

Sequencing mapping for SNP identification analysis was performed using CLC Genomics Workbench (version 5.5.2; CLC bio, Aarhus, Denmark). Trimmed sequence reads were aligned against the Trinity assembly contigs. Mapping of reads from each group to the reference sequence was performed with mismatch cost of 2, deletion cost of 3 and insertion cost of 3. The highest scoring matches that shared ≥ 95% similarity with the reference sequence across ≥ 90% of their length should be included in the alignment. The non-unique mappings were removed. Finally, all mapping results were converted to BAM format.

The initial SNP identification was conducted by the rmdup and mpileup function of SAMtools version 0.1.18 [60]. PoPoolation2 version 1.201 [61] was used to call the genotype at each variant with the lowest criteria setting in order to get all possible real SNPs, SNP loci that has more than 2 allele variants were discarded. Two factors that are important for increasing quality of putative SNPs were set based on the sequence error rate and total coverage: 1) minimum reads in each group ≥ 6, and 2) Total minor allele reads count >3. SNPs that passed all the optimal factors were considered as initial SNPs.

Significant SNPs were identified between resistant catfish group and susceptible catfish group. SNPs which displayed heterozygous genotype (allele variants = 2 and minor allele reads ≥2 in each group) were retrieved to test the difference level of allele frequencies between resistant catfish group and susceptible catfish group using two-tailed Fisher’s Exact test [62]. The threshold was set as FDR p-value ≤0.05.

### Analysis of bulk frequency ratios and allele specific expression

In order to compare the SNP allele frequencies more directly, bulk frequency ratios (BFR) were generated from the RNA-seq data between the two bulks, the resistant fish and the susceptible fish, as follows: the frequency of informative base (the major allele of resistant fish) was first calculated for resistant and susceptible bulks and then BFR was equal to the ratio of these frequencies between the bulks. The ratio of the two alleles was also analyzed with combined bulks of both resistance and susceptible fish (i.e. total count of one allele in both resistant and susceptible group divided by total count of the other allele) to help classify the genes containing significant SNPs into several categories: 1) The genes containing significant SNPs with BFR ≥ 4 and allele ratio ≤ 9; 2) The genes containing significant SNPs with BFR ≥ 4 and allele ratio ≥ 14; 3) The genes containing significant SNPs with BFR ≤ 4 and allele ratio ≥ 14; 4) The genes containing significant SNPs with BFR ≤ 4 and allele ratio ≤ 9; 5)The genes containing significant SNPs with allele ratio from 9 to 14. The genes with BFR ≥4 and allele ratio ≤ 9 were defined as segregation involved candidate genes, the genes with BFR <4 and allele ratio ≥ 14 were defined as allele specific expression (ASE) involved candidate genes, and the genes with BFR ≥4 and allele ratio ≥ 14 were defined as both segregation and ASE involved genes. An extra fisher’s exact test were used to check the significant different level between the two alleles on the genes with allele ratio ≥ 14, by setting the expect allele ration equal to 7:1 and FDR adjusted p-value ≤ 0.05. Analysis of parental origin of the alleles for the genes with high allele ratio was then performed. The inter-species SNPs database of blue and channel catfish [63] and the parents’ genotype of two target families on SNP chip (unpublished data) were used. The inter-species SNPs were mapped to the genes containing significant SNPs with high allele ratio (allele ratio > 14) to check whether they shared the same position with the significant SNPs. The major allele origin of mapped SNPs was labeled based on the genotype information of the inter-specific SNPs.

### Genomic location of ESC resistance-related genes

Genes harboring significant SNPs with BFR ≥5 and combined allele ratio ≤4 were used as query to map to the whole genome scaffold and linkage groups (unpublished data) by BLASTN with e-value of 1e^-20^. The mapped scaffolds were then located to the linkage groups by 2^nd^ generation catfish linkage map [33]. The linkage groups contain more than 10 genes with significant SNPs and at least one gene harboring significant SNPs with BFR ≥ 10 were identified as potential genomic regions harboring candidate genes for ESC resistance.

### Availability of Supporting Data

Raw RNA-seq reads data supporting the results of this article are available in the NCBI Sequence Read Archive (SRA) under Accession SRP028159 (http://www.ncbi.nlm.nih.gov/sra).

## Competing interests

The authors declare that they have no competing interests.

## Authors’ contributions

RW, LS and LB conducted the major part of the research including preparation of the samples, bioinformatics analysis and manuscript preparation. JZ, YJ, JY, LS, and SL were involved in one or more processes of RNA extraction or bioinformatics analysis. JF prepared tissue samples for this work. ZL conceived, designed and guided the research and involved in manuscript preparation. All authors read and approved the final manuscript.

## Supplementary Material

Additional file 1: Table S1 Differentially expressed genes between the resistant and susceptible fish. Click here for file

Additional file 2: Table S2 Genes containing significant SNPs. Click here for file

Additional file 3: Table S3 BFR and combined allele ratios of genes containing significant SNPs. Click here for file

Additional file 4: Table S4 Allele-specific expressed genes. Click here for file
